# Supplementary material for: The causal role of circulating inflammatory markers in osteoporosis: a bidirectional Mendelian randomized study
Source: Front Immunol. 2024 Jul 18;15:1412298. doi: 10.3389/fimmu.2024.1412298 (PMC11291241; doi:10.3389/fimmu.2024.1412298)
Supplement: Supplementary file 1 [file DataSheet_1.zip › supplementary material/OSTEOPOROSIS-CIM/GCST90274832.tsv.gz_forest.pdf]

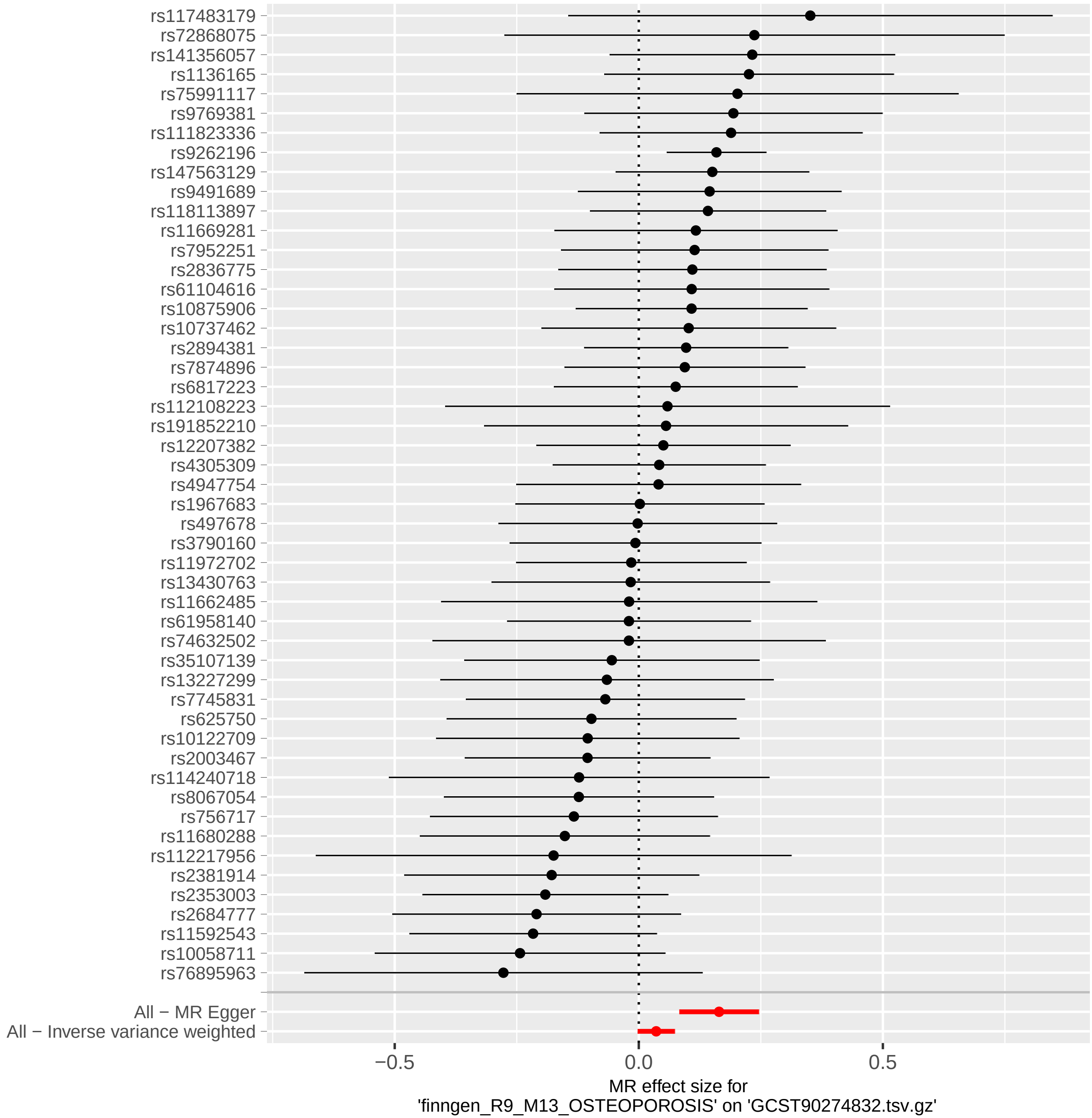
rs117483179
rs72868075
rs141356057
rs1136165
rs75991117
rs9769381
rs111823336
rs9262196
rs147563129
rs9491689
rs118113897
rs11669281
rs7952251
rs2836775
rs61104616
rs10875906
rs10737462
rs2894381
rs7874896
rs6817223
rs112108223
rs191852210
rs12207382
rs4305309
rs4947754
rs1967683
rs497678
rs3790160
rs11972702
rs13430763
rs11662485
rs61958140
rs74632502
rs35107139
rs13227299
rs7745831
rs625750
rs10122709
rs2003467
rs114240718
rs8067054
rs756717
rs11680288
rs112217956
rs2381914
rs2353003
rs2684777
rs11592543
rs10058711
rs76895963
All − MR Egger
All − Inverse variance weighted
−0.5
0.0
0.5
MR effect size for
'finngen_R9_M13_OSTEOPOROSIS' on 'GCST90274832.tsv.gz'
